# Understanding the Role of Lithium Doping in Reducing Nonradiative Loss in Lead Halide Perovskites

**DOI:** 10.1002/advs.201800736

**Published:** 2018-10-23

**Authors:** Zhishan Fang, Haiping He, Lu Gan, Jing Li, Zhizhen Ye

**Affiliations:** ^1^ State Key Laboratory of Silicon Materials School of Materials Science and Engineering Zhejiang University Hangzhou 310027 China

**Keywords:** doping, perovskites, photoluminescence, recombination

## Abstract

Adding alkali metal into lead halide perovskites has recently been demonstrated as an effective strategy for reducing nonradiative loss. However, the suggested role of the alkali metal is usually limited to surface passivation, and the semiconductor doping effect is rarely discussed. Here, the mechanism of lithium doping in the photocarrier recombination in solution‐processed methylammonium lead halide films is investigated by photoluminescence and photoelectron spectroscopies. It is demonstrated that lithium doping weakens the electron–phonon coupling and acts as donor in perovskites, which provide solid evidence that lithium enters the lattice rather than just in the surface region. The n‐type doping creates free electrons to fill the trap states in both the bulk and surface regions, leading to suppressed trapping of photocarriers and reduces nonradiative recombination.

## 1 Introduction

Solution‐processed lead halide perovskites have attracted extensive attentions in recent years due to their outstanding photoelectric properties and excellent performance in photovoltaic^1^, ^2^, ^3^, ^4^, ^5^ and light‐emitting devices.^6^, ^7^, ^8^, ^9^, ^10^, ^11^ Perovskite solar cells with power conversion efficiency of 23.3%^12^ and light‐emitting diodes (LEDs) with external quantum efficiency of 16.3%^13^ have recently been reported. Nevertheless, trap states with typical density of ≈10^15^–10^16^ cm^−3^, which is comparable to the density of photogenerated or photoinjected carriers in solar cell or LEDs under device working conditions, are normally found in solution‐processed perovskite films.^14^, ^15^ The trapping of carriers in trap states and subsequent nonradiative recombination result in relatively low photoluminescence quantum yield, especially in methylammonium lead iodide perovskite (MAPbI_3_) perovskite films. Therefore, modification of materials is desired for further improving the luminescent properties and device performance.

General strategies for reducing the nonradiative recombination are surface passivation and doping. Surface passivation has been extensively studied^16^, ^17^ by treating or coating the perovskite films with various chemicals. However, this strategy is difficult to reach trap states in the bulk. In fact, in solution‐processed perovskite films the bulk trap density could be comparable to the surface/interfacial one.^18^ In this regard, doping, an effective way to modify the properties of semiconductors, appears to be hopeful because it works in both the surface/interfacial and the bulk region. Recently, alkali metal doping was demonstrated as an effective way to reduce nonradiative loss in perovskites.^19^, ^20^ Nevertheless, the role of alkali metal is usually suggested as passivating the surface and grain boundaries. This means that the alkali metal in these cases play a role of additive rather than semiconductor doping in the true sense. In principle, semiconductor doping refers to the incorporation of dopant atoms into the host lattice, either in substitutional or interstitial sites. In this respect, so far many works claiming doping in perovskites actually dealt with additives.

There were a few reports on the lithium doping of lead halide perovskites.^21^, ^22^, ^23^, ^24^, ^25^ Cao et al.^25^ studied the incorporation of extrinsic alkali cations (including Li) into perovskites both experimentally and theoretically, and found that the alkali cations occupy interstitial sites, resulting in suppression of iodine ion migration. The interstitial occupation also indicates that Li can act as n‐type dopant and introduce energy level in the bandgap. Indeed, conductivity enhancement was recently reported in Li‐doped perovskites.^24^, ^26^ It has been shown that Li‐doping may have various effects on the structural,^25^ electrical,^26^ magnetic,^24^ and optical absorption^24^ properties of perovskites. However, the role of lithium in the carrier recombination processes as well as its mechanisms, which is very important to understanding the photophysics in doped perovskites, has not been addressed.

In this work, we dope lead halide perovskites by lithium ions and focus on the mechanism of lithium doping on the photocarrier recombination. By analyzing the thermal quenching of photoluminescence (PL), we show that the ratio of nonradiative recombination is significantly reduced upon lithium doping. The shift of Fermi level toward the conduction band reveals that lithium act as donors, which leads us to propose a model based on passivation of the carrier traps through state filling to account for the improved PL. Our results thus suggest a role other than surface passivation played by alkali metal in perovskites.

## 2 Results and Discussion

**Figure 1:**
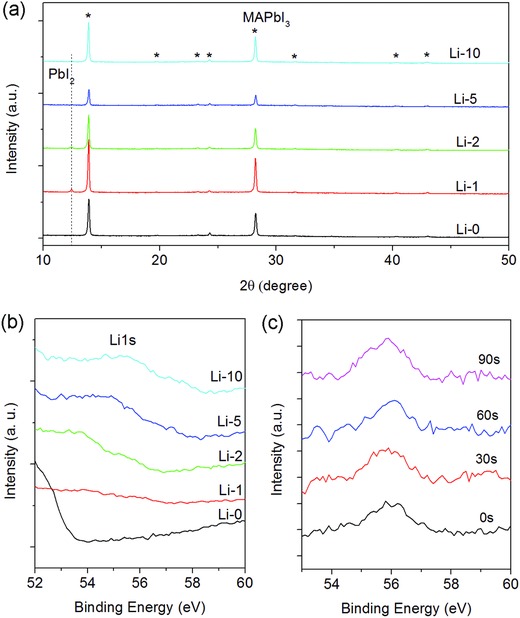
a) XRD patterns of a series of Li‐doped MAPbI_3_ films. The peaks labeled by asterisk can be indexed to tetragonal perovskite. Dashed line represents the diffraction of PbI_2_. b) XPS Li1s core level spectra of a series of Li‐doped MAPbI_3_ films. c) XPS Li1s core level spectra of the Li‐10 sample after Ar^+^ ion etching for 30, 60, and 90 s. The curves are vertically separated for clarity.

Solution‐processed MAPbI_3_ was used to study the effects of Li doping. Doping of lithium was realized by adding certain amount of LiI into the precursor solution. The films were formed following the reported method^27^ on glass substrate with a layer of pre‐deposited polyvinylpyrrolidone (PVP) to enable better film formation with smooth and compact morphology (Figure S1, Supporting Information). The experimental details for the growth can be found in Experimental Section. A series of samples with different LiI content (0%, 1%, 2%, 5%, and 10%) in the precursor solution, were labeled as Li‐0, Li‐1, Li‐2, Li‐5, and Li‐10, respectively. As revealed by the XRD patterns in **Figure** **1**a, all the doped samples show the same set of diffraction peaks indexed to tetragonal perovskite.^28^ For Li‐1 and Li‐2 samples a tiny peak from PbI_2_ is detected, which has been frequently reported in MAPbI_3_ films.^29^ The diffraction peaks show very close width for all the samples (Figure S2, Supporting Information), indicating that the doping does not result in obvious change in grain size, in contrast to the case of Na or K doping reported in the literatures.^19^ The incorporation of Li is testified by measuring the core level X‐ray photoelectron spectroscopy (XPS) spectra of Li1s. Since XPS only collects information on the surface, measurements were also performed after the samples were etched by Ar^+^ ion in order to obtain information in the bulk region. As shown in Figure 1b,c, with increasing Li content in the precursor or etching time from the film surface, Li1s signal around 55 eV can be discriminated in both the pristine and etched samples, revealing the incorporation of Li in both the surface and bulk regions of MAPbI_3_ perovskites. Nevertheless, it is difficult to determine the Li content in the films quantitatively due to the very low sensitivity factor of Li. The signal splits into a main peak and a shoulder (more clearly seen in the Li‐2 sample), which may be assigned to interstitial Li and bonded Li, respectively,^30^ suggesting the coexistence of Li atoms siting in different sites. This is consistent with recent density functional theory calculations,^25^ which revealed that for Li atoms in CH_3_NH_3_PbI_3_ perovskite both interstitial and A‐site (CH_3_NH_3_^+^) substitutional occupations are thermodynamically possible, while at low Li concentration the interstitial Li is more favorable due to the lower formation energy. It should be noted that at low Li concentration the interstitial Li in MAPbI_3_ is expected to be energetically stable due to the larger MAPbI_3_ unit cell and less distortion of PbI_6_ octahedral when compared with other halide perovskites.^22^ In fact, in our experiments the incorporation of Li does not induce additional conversion reactions such as the formation of metallic Pb as reported previously for the high doping case,^22^ as shown in the almost identical core level spectra of Pb4f, I3d, and N1s before and after Li doping (Figure S3, Supporting Information). The reasonable stability of Li‐doped perovskites is also supported by the aging effect of PL, which does not show obvious difference from that of the undoped sample (Figure S4, Supporting Information).

**Figure 2:**
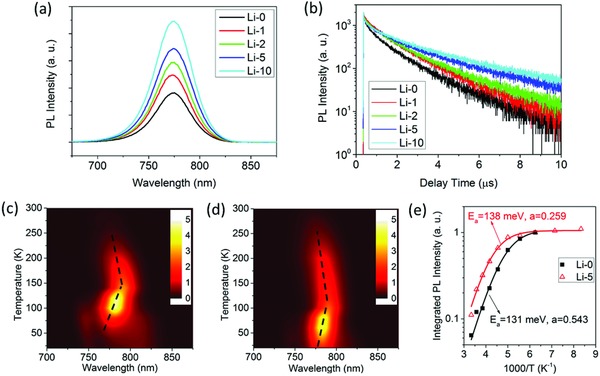
PL properties of the Li‐doped MAPbI_3_ films. a) Steady‐state room temperature PL spectra. b) PL decay traces. Temperature‐dependent PL spectra of the c) undoped and d) Li‐5 perovskite films. Dashed lines represent the evolution of PL peak wavelengths. e) Arrhenius plot of the thermal quenching of PL intensity. Solid lines represent the fitting results using Equation (1).

**Figure** **2** shows the PL properties of the Li‐doped perovskites. With increasing Li doping, the room temperature PL intensity gradually increases by up to 2.6 folds (Figure 2a). Meanwhile, the PL lifetime also shows monotonous increase with increasing Li doping (Figure 2b). It should be noted that in MAPbI_3_ perovskite the recombination is usually considered to be bimolecular. However, the solution‐processed perovskite films typically have a trap density of ≈10^16^ cm^−3^,^18^ which means that at low excitation fluence the photodoping effect plays an important role and the trap‐assisted recombination should be characterized by monoexponential decay.^31^ In our experiments, the excitation fluence is low (≈4 nJ cm^−2^), and the corresponding photocarrier density (≈5 × 10^14^ cm^−3^) is much lower than the typical trap density. In fact, as will be discussed later, the excitation density‐dependent PL results (Figure 4a) reveal that the nature of dominant recombination in our experiments is trap‐assisted rather than bimolecular. Moreover, it was found that bimolecular fit cannot well reproduce the experimental results (Figure S5, Supporting Information). Therefore, monoexponential decay was used to analyze the PL decay traces. According to the work of deQuilettes et al.,^32^ stretched exponential decay was adopted to account for the energetic disorder due to the polycrystalline nature of the films. The fitting results reveal a slow process with long lifetimes exceeding 1 µs (Figure S5 and Table S1, Supporting Information). Keeping in mind that PL lifetime represents the effective lifetime that contains both radiative (τ_r_) and nonradiative lifetime (τ_nr_) as 1/τ_PL_ = 1/τ_r_ + 1/τ_nr_, the prolonged PL decay suggests reduced nonradiative recombination. In order to acquire more information about the nonradiative recombination, we performed temperature‐dependent PL spectra because the thermal quenching of PL is generally governed by the thermal activation of nonradiative channels (whether they correspond to level depopulation or to the activation of a nonradiative recombination center). A widely used expression for the PL intensity as a function of temperature is^33^, ^34^

(1) $$I\left(T\right)=\frac{I_{0}}{1+aT^{\frac{3}{2}}\exp\left(\frac{-E_{a}}{k_{B}T}\right)}$$

where *I*_0_ is the PL intensity at low temperature limit, *E_a_* is the activation energy of nonradiative channel, *a* represents the weight for the probability of nonradiative channel, and *k_B_* is the Boltzmann constant. Note that the expression contains a *T* ^3/2^ term accounting for the temperature‐dependent radiative lifetime for carriers/excitons in 3D structure,^35^ which is usually omitted for simplification. It is unclear so far that whether the thermal activation of carrier‐trapping process or the increase of defects density at higher temperature (note the point defects are generated thermally which is a continual equilibrium process) is responsible for the PL quenching of perovskites. Nevertheless, for both cases the model of Equation (1) is appropriate to describe the PL quenching, and such a model has been extensively adopted in studying the PL quenching of lead halide perovskites.^17^, ^36^, ^37^, ^38^, ^39^, ^40^, ^41^ For MAPbI_3_, there is a phase transition around 156 K.^42^ Therefore, data for temperature above the phase transition are used in Equation (1). As shown in Figure 2c, the undoped perovskite shows a phase transition around 150 K as evidenced by the kink in the temperature‐dependent PL peak wavelength, which is consistent with previous reports for MAPbI_3_. While for Li‐doped sample (Figure 2d), interestingly, the kink occurs around 100 K, suggesting a lower phase transition temperature. The Li‐doped perovskite film also shows a different PL quenching behavior from the undoped one. As shown in Figure 2e, the integrated PL intensity of the Li‐doped film quenches slower than the undoped one. Fitting the two sets of data with Equation (1) gives rise to similar activation energies (≈130–140 meV) for both films, indicating the same nonradiative channel responsible for the PL quenching.

Although the Li‐doped and undoped films reveal the same nonradiative channel for photocarrier recombination, the Li‐doped film shows a much smaller weight for probability (*a* = 0.259 for doped and 0.543 for undoped film), indicating a reduced nonradiative loss. As a result, the relative internal quantum efficiency,^43^ defined as the ratio of PL intensity at certain temperature to the one at low temperature limit (in this case the phase transition temperature), is enhanced by a factor of ≈2 at room temperature after Li‐doping.

**Figure 3:**
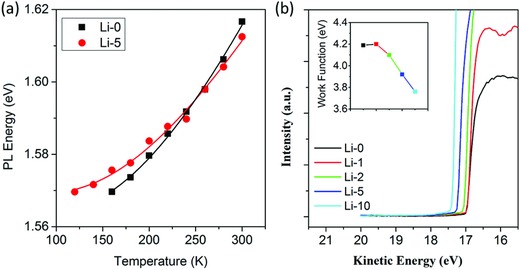
a) Temperature‐dependent PL energy of the undoped (Li‐0) and Li‐doped (Li‐5) MAPbI_3_ films. Solid lines represent fitting curves using Equation (2). b) UPS spectra of a series of Li‐doped MAPbI_3_ films. Inset shows the derived work function using the energy of He I (21.2 eV) as the reference.

Since surface/grain boundary passivation has been shown to reduce the nonradiative loss in perovskites,^19^, ^20^ it is essential to determine whether the doped Li enters into the lattice or just stays in the surface/grain boundary region to unravel the actual role of Li doping. It is difficult to directly visualize the spatial distribution of Li in the films by elemental mapping due to its very light weight and the lack of effective analytical tools. Nevertheless, spectroscopies may provide convincing evidences. **Figure** **3**a shows the temperature‐dependent PL peak energy of the tetragonal MAPbI_3_, which can be taken as the bandgap energy. One can find that the doping of Li clearly changes the temperature dependence of the bandgap. It has been known for a long time that the temperature dependence of semiconductor bandgaps is mainly determined by temperature‐dependent electron–phonon interactions. This is because the bandgap reflects the bond energy, while the lattice phonons are easily excited in large numbers at moderate temperature due to their small energies, which influence the bonding through various orders of interactions.^44^ The data in Figure 3a can be fitted by the well‐established phenomenological expression for temperature‐dependent bandgap^44^, ^45^

(2) $$E_{g}\;\left(T\right)=E_{g}\;\left(0\right)+S\left\langle E_{\mathrm{ph}}\right\rangle \left[\coth\left(\left\langle E_{\mathrm{ph}}\right\rangle /2k_{B}T\right)-1\right]$$

where 〈*E*_ph_〉 is an average phonon energy, *S* is a dimensionless electron–phonon coupling constant, and *k_B_* is the Boltzmann constant. Note that for lead halide perovskites the bandgap decreases with decreasing temperature, which is contrary to most semiconductors. Thus the second term in Equation (2) is used with the plus sign,^45^ rather than the minus sign in most semiconductors. The fit of the PL peak using Equation (2) gives *S* values of 3.87 and 2.93 for Li‐0 and Li‐5 films, respectively. The results indicate the obviously reduced electron–phonon coupling in MAPbI_3_ perovskite after Li‐doping. Since the electron–phonon coupling is an intrinsic process involving all the lattice phonons, the change of *S* value thus strongly suggest that the doped Li enters into the perovskite lattice. Another evidence for the conclusion comes from the change of work function derived from ultraviolet photoelectron spectroscopy (UPS) measurements. As shown in Figure 3b, the undoped perovskite film shows a work function of 4.2 eV. With increasing Li doping, the work function decreases monotonously, reaching 3.76 eV for the Li‐10 film. The significant change of the work function is a result of upward shift of the Fermi level, indicating n‐type doping of lithium. This is not surprising because the size of lithium ions is very small so they can sit in interstitial sites, acting as donors. The n‐type doping effect of Li in perovskites has been reported by Jiang et al. recently.^26^

**Figure 4:**
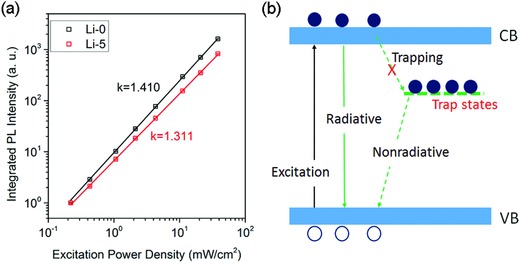
a) Log–log plot of the PL intensity as a function of excitation power. The decrease of slope indicates the reduced density of empty trap states. b) Schematic to illustrate recombination mechanisms of the Li‐doped MAPbI_3_ films. The doping of Li creates free carriers to fill the trap states, thus suppressing the trapping of photocarriers and improving the radiative recombination.

It has been demonstrated in organic semiconductors that doping may passivate the trap states by carrier filling.^46^ To test whether this also works in our Li‐doped perovskites, we carried out excitation density–dependent PL measurements (Figure S6, Supporting Information). **Figure** **4**a shows the integrated PL intensity as a function of excitation power density of the undoped (Li‐0) and Li‐doped (Li‐5) perovskite films. Both samples show power‐dependence *I*_PL_ ∼ *I*_ex_ *^k^* with *k* = 1.410 and 1.311, respectively. According to a model considering the trap‐assisted recombination proposed by Saba et al.,^47^ *k* should equal to 1.5 when the majority of trap states are unoccupied, and it gradually changes from 1.5 to 1 when the trap states are filled. The decrease of *k* from 1.426 to 1.307 in our samples thus indicates the filling of trap states by free electrons produced by n‐type Li doping. This is also supported by the evolution of PL intensity with the illumination time. As shown in Figure S7 in the Supporting Information, the undoped film shows gradually increased PL intensity with increasing illumination time, a result of trap filling by photocarriers. After Li‐doping, such filling effect is significantly suppressed.

Based on above results, we propose a model for the photocarrier recombination in Li‐doped perovskites considering the contribution of trap states, as shown in Figure 4b. Note that in our experiments the excitation level is relatively low, corresponding to a photocarrier concentration of ≈10^14^ cm^−3^, which is lower than typically reported trap density in solution‐processed MAPbI_3_ films. For undoped film, the photogenerated electrons will be quickly trapped by the trap states followed by nonradiative recombination with free holes, leading to weak PL. After Li‐doping, however, there exist free electrons even in dark, which passivate a part of trap by filling the states before photoexcitation. Thus the trapping of photocarriers is suppressed, leading to enhanced radiative recombination. It should be noted that such a mechanism based on semiconductor doping works not only for surface traps but also for bulk traps.

## 3 Conclusions

In summary, we have grown Li‐doped MAPbI_3_ films by a solution process and investigated the mechanism of Li‐doping in the carrier recombination by comprehensive PL and UPS studies. Li doping results in weakened electron–phonon coupling and n‐type doping, indicating that Li enters into the perovskite lattice, rather than sits just on the surface or grain boundaries. We found that the doping of Li leads to enhanced PL intensity and prolonged PL lifetime. Temperature‐dependent PL indicates that the reduced nonradiative recombination is responsible for the improved PL properties. The reduction of nonradiative recombination is a result of n‐type doping and the subsequent trap filling. Our results suggest a role of carrier doping besides the frequently invoked surface passivation played by alkali metal in lead halide perovskites, which we believe may provide insights into the mechanisms of doping in engineering the carrier recombination dynamics.

## 4 Experimental Section

*Chemicals*: Lead(II) iodide (PbI_2_, 99.999%) and Lithium iodide (LiI, 99.999%) were obtained from Sigma‐Aldrich. *N*,*N*‐dimethylformamide (DMF, ≥99.9%) and PVP (M.W. 1 300 000) was purchased from Alfa Aesar. CH_3_NH_3_I (MAI, 99.5%) was bought from Xi'an p‐OLED Corp. All the chemicals were used as‐received.

*Preparation of CH_3_NH_3_PbI_3_ Film*: Glass substrates (2 cm × 2 cm) were cleaned by acetone, ethanol, and deionized water, and then modified their surface with ozone–ultraviolet treatment. A series of 3:1 molar ratio of (MAI + LiI):PbI_2_ was dissolved in DMF to form a 0.88 m solution, as Li:Pb = 0%, 0.5%, 1%, 2%, 5%, and 10% in molar ratio. Prior to the perovskite film deposition, the substrate was interface‐modified by PVP. 0.5 wt% PVP solution in DMF was spin coated onto glass at 2000 rpm for 1 min, and then annealed at 150 °C for 15 min to induce cross‐linking. The precursor solutions were then spin coated onto the glass substrates at 3000 rpm for 40 s, then annealed at 120 °C for 10 min.

*Characterizations*: The MAPbI_3_ film was characterized by powder XRD using an X'pert PRO diffractometer (PANalytical) with Cu Kα radiation (λ = 0.15406 nm). XPS measurements were carried out on a Kratos Axis Supra (Kratos Analytical Ltd.) spectrometer using Al Kα radiation (1486.6 eV) as the excitation source. Etching was carried out using an argon ion gun as the ion source. A low‐energy (500 eV) Ar^+^ ion beam was used to reduce as much as possible ion beam damages and preserve chemical information. The UPS was also performed on Kratos Axis Supra spectrometer using He I (21.2 eV) as the radiation source. Scanning electron microscope images were carried out using a Hitachi S4800 operating at an acceleration voltage of 5 kV. The steady‐state and time‐resolved photoluminescence were measured on a FLS 920 fluorescence spectrometer (Edinburgh Instrument), and a 405 nm laser with a wavelength of 404.2 nm and pulse width of 58.6 ps was used as the excitation source. The excitation fluence is ≈4 nJ cm^−2^. The PL decay data were recorded using time‐correlated single photon counting technique. For excitation density–dependent measurements, the light fluence was tuned by neutral attenuators. For temperature‐dependent measurements, the samples were placed in a closed‐cycle helium cryostat.

## Conflict of Interest

The authors declare no conflict of interest.

## Supporting information

Supplementary
