# Supplementary material for: Understanding the Role of Lithium Doping in Reducing Nonradiative Loss in Lead Halide Perovskites
Source: Adv Sci (Weinh). 2018 Oct 23;5(12):1800736. doi: 10.1002/advs.201800736 (PMC6299680; doi:10.1002/advs.201800736)
Supplement: Supplementary file 1 — Supplementary [file ADVS-5-1800736-s001.pdf]

Copyright WILEY-VCH Verlag GmbH & Co. KGaA, 69469 Weinheim, Germany, 2018.

ADVANCED SCIENCE
Open Access

Supporting Information

for *Adv. Sci.,* DOI: 10.1002/advs.201800736

Understanding the Role of Lithium Doping in Reducing Nonradiative Loss in Lead Halide Perovskites

Zhishan Fang, Haiping He, Lu Gan, Jing Li, and Zhizhen Ye*

Supporting Information

Understanding the Role of Lithium Doping in Reducing Nonradiative Loss in Lead Halide Perovskites

Zhishan Fang, Haiping He, Lu Gan, Jing Li and Zhizhen Ye*

State Key Laboratory of Silicon Materials, School of Materials Science and Engineering, Zhejiang University, Hangzhou 310027, China

*Corresponding author. Email: hphe@zju.edu.cn

Table S1. Fitting results of the PL lifetimes with stretched-exponential decay $I(t) = I_0 e^{-(t/\tau_c)^\beta}$. The average lifetime[1] is defined as $< \tau > = \frac{\tau_c}{\beta}\Gamma(\frac{1}{\beta})$.

	Li-0	Li-1	Li-2	Li-5	Li-10
τ_c (ns)	357.3	602.7	632.9	593.2	649.4
β	0.506	0.646	0.564	0.466	0.449
<τ> (ns)	705.1	829.3	1038.2	1363.1	1617.6

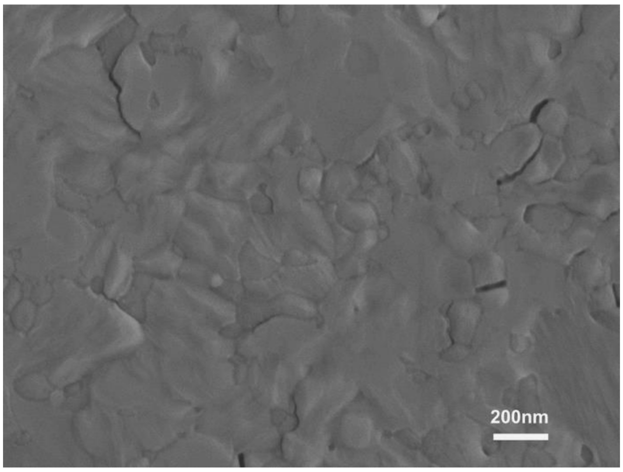

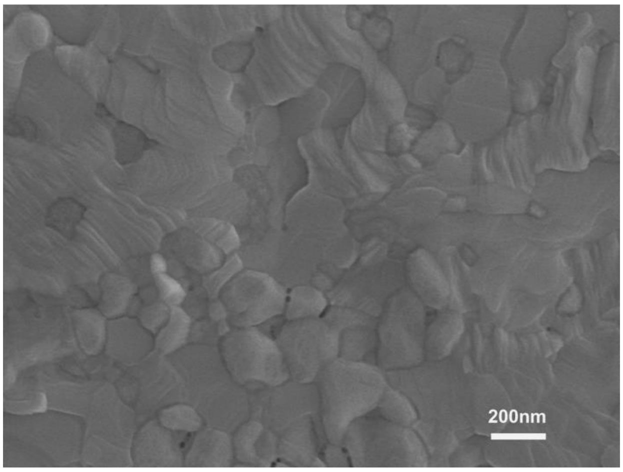

Figure S1. SEM images of the undoped (Li-0, top) and Li-doped (Li-5, bottom) $MAPbI_3$ films, showing the smooth and compact morphology.

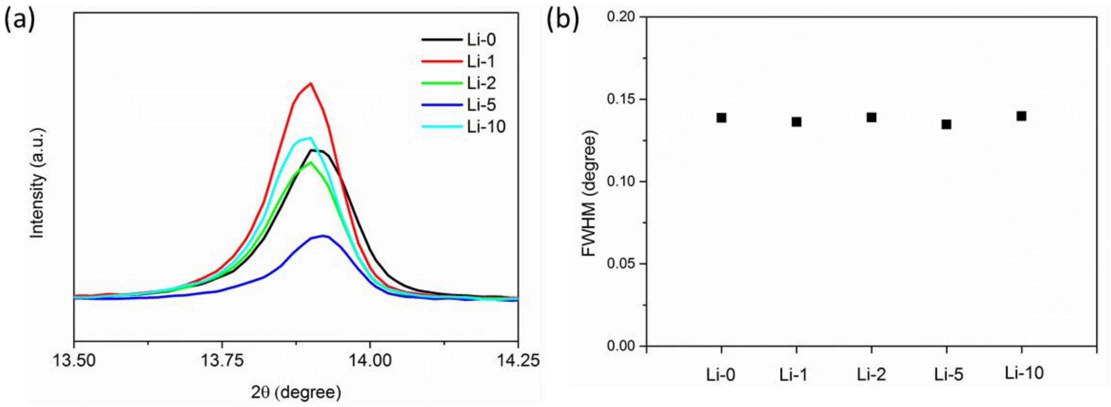

Figure S2. (a) Magnified XRD patterns and (b) FWHM (full width at half maximum) of the (110) plane of a series of Li-doped $MAPbI_3$ films. The FWHM keeps almost constant for all the doping contents, indicating unchanged grain size.

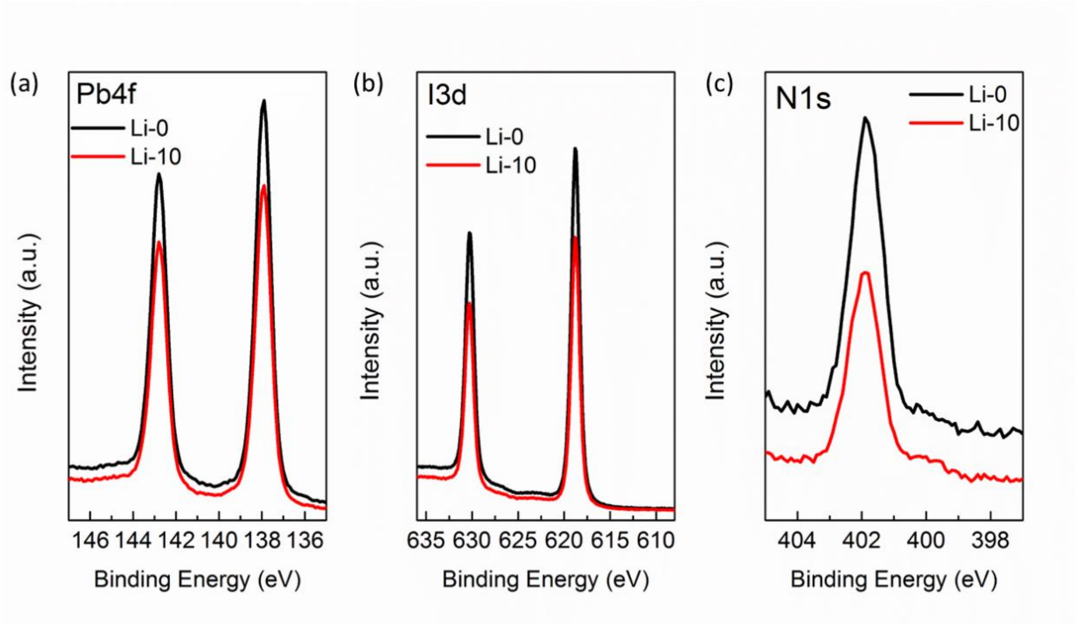

Figure S3. XPS core level spectra of Li-0 and Li-10 films. (a) Pb 4f, (b) I3d, (c) N1s. The Li doping does not changes the peak energy and lineshape of the spectra.

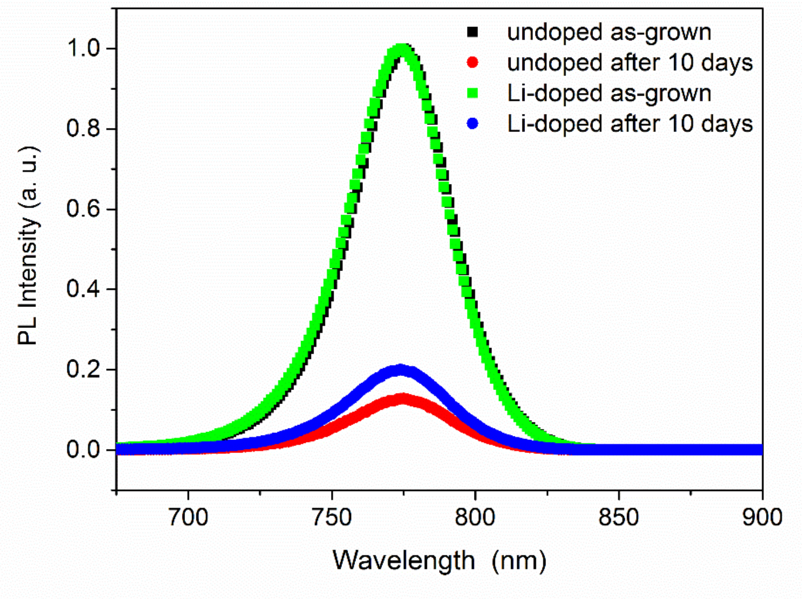

Figure S4. PL spectra of the undoped and Li-doped perovskite films before and after aging for 10 days. The aging was performed by exposing the films to air with 50% humidity. The PL intensity of the aged samples are normalized to the as-grown ones.

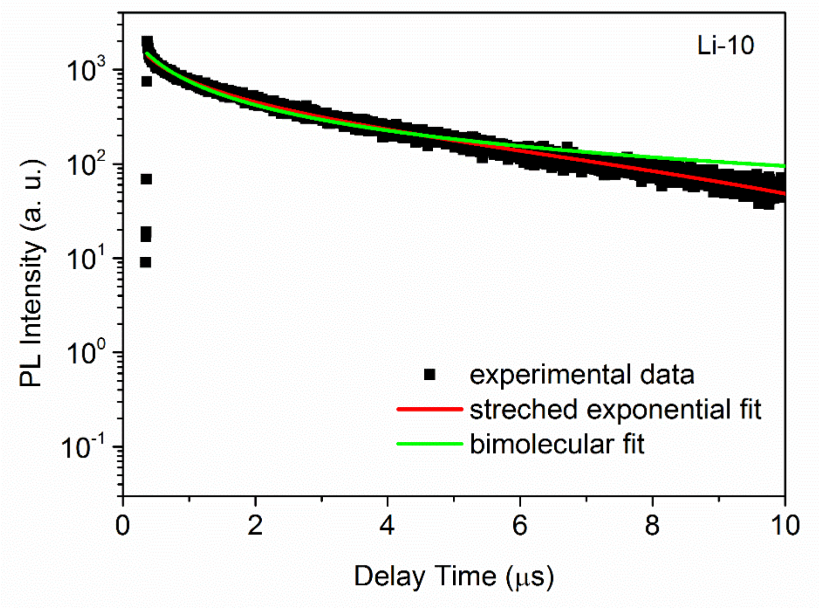

Figure S5. Stretched-exponential (red) and bimolecular (green) fit of the decay trace of Li-doped (Li-10) $MAPbI_3$ film. The bimolecular fit cannot well reproduce the experimental data.

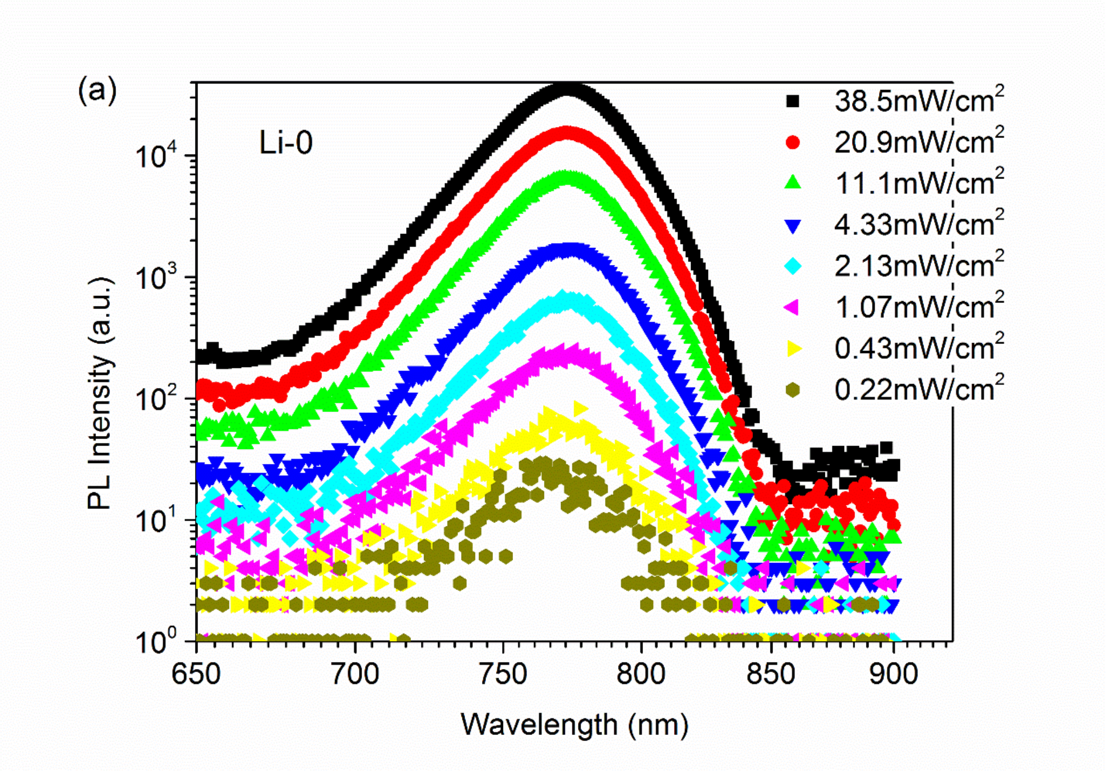

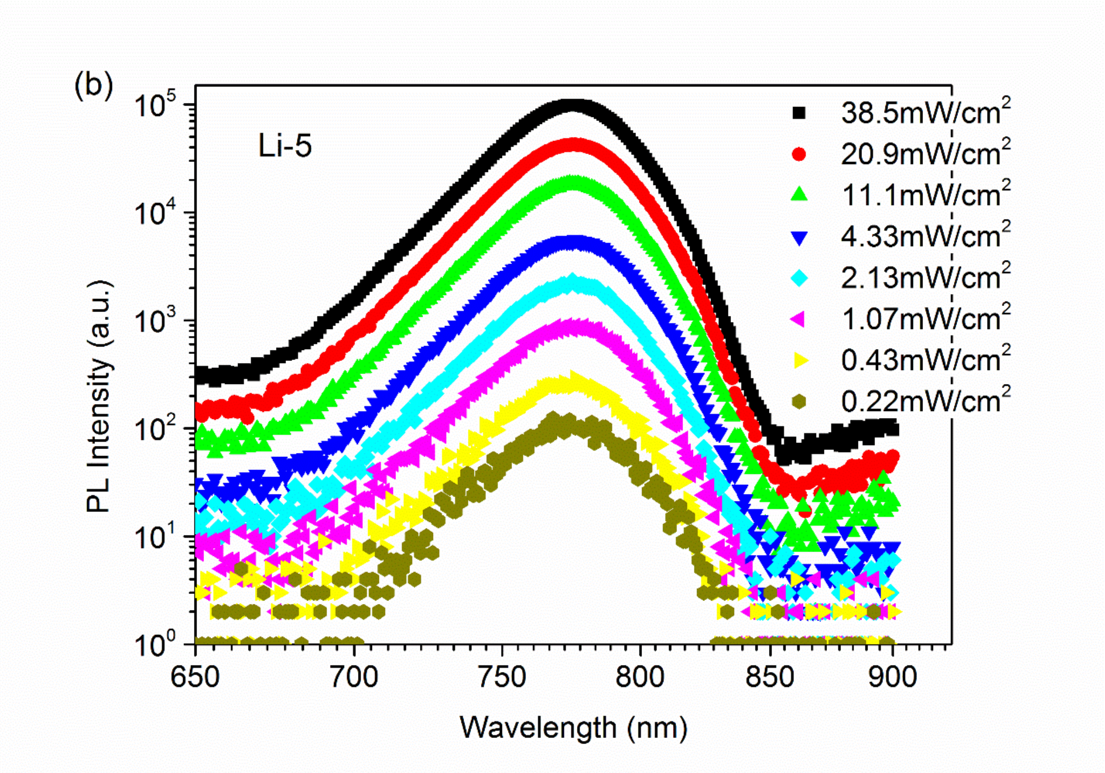

Figure S6. PL spectra under various excitation power densities. (a) Undoped (Li-0) $MAPbI_3$ film, (b) Li-doped (Li-5) $MAPbI_3$ film.

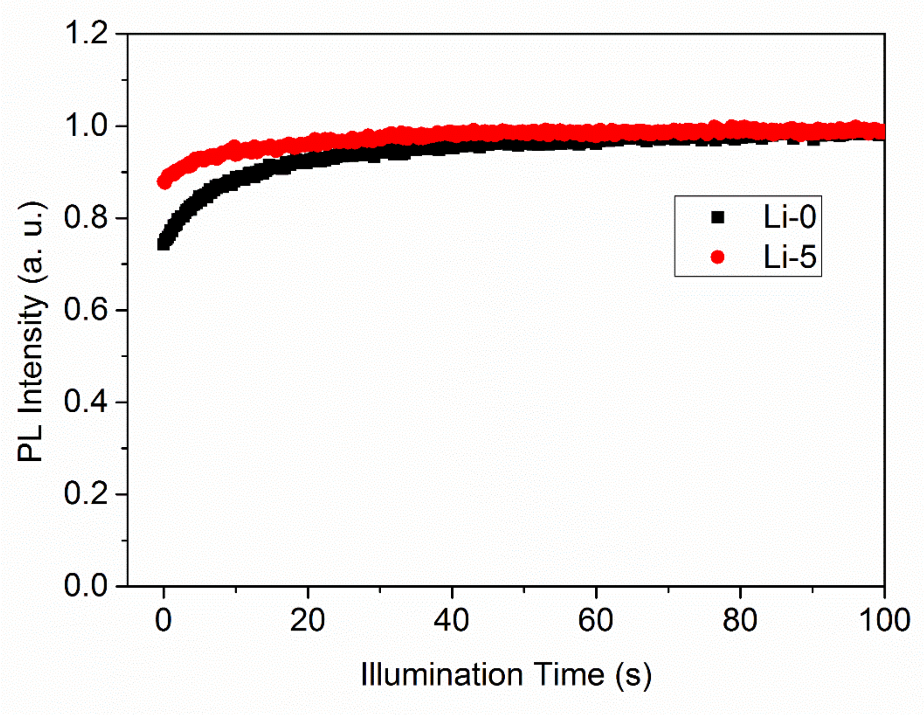

Figure S7. Time evolution of PL intensity of the undoped and Li-doped $MAPbI_3$ films

with continuous illumination of the excitation light.

Reference

1. D. W. deQuilettes, S. M. Vorpahl, S. D. Stranks, H. Nagaoka, G. E. Eperon, M. E. Ziffer, H. J. Snaith, D. S. Ginger, *Science* **2015**, 10.1126/science.aaa5333.
